# A Bibliometric Analysis of Leprosy during 2000–2021 from Web of Science Database

**DOI:** 10.3390/ijerph19148234

**Published:** 2022-07-06

**Authors:** Xiang Li, Jing Yang, Lianhua Zhang, Guangjie Jin, Li Xu, Fujin Fang, Yunhui Li, Pingmin Wei

**Affiliations:** 1 Department of Epidemiology and Health Statistics, School of Public Health, Southeast University, Nanjing 210009, China; 230218457@seu.edu.cn (X.L.); 220203877@seu.edu.cn (J.Y.); 220214010@seu.edu.cn (L.X.); 230218461@seu.edu.cn (F.F.); yhli@seu.edu.cn (Y.L.); 2 Department of Chronic Infectious Disease Control and Prevention, Jiangsu Provincial Center for Disease Control and Prevention, Nanjing 210000, China; zhanglh@jscdc.cn (L.Z.); jingj@jscdc.cn (G.J.)

**Keywords:** leprosy, bibliometrics, early diagnosis, stigma

## Abstract

In recent years, after the essential elimination of leprosy (the prevalence of which is <1/100,000), the trends, research hotpots, and frontiers of leprosy research are not clear. This study provides a detailed overview of leprosy in terms of papers, journal, language, year, citations, h-index, author keywords, institution, and country through bibliometrics. The results are as follows: (1) The publication rate has increased in recent years, and 8892 papers were obtained. Most of the publications are in English, and the subject categories are mainly focused on “Dermatology.” The “*leprosy review*” published the most significant number of papers on leprosy, followed by “*Plos Neglected Tropical Disease*” and “*International Journal of Leprosy and Other Mycobacterial Diseases*.” (2) Leprosy-related research was contributed to by 24,672 authors, and the ten authors with the most significant number of publications were identified. (3) The University of London (including the London School of Hygiene and Tropical Medicine) has the highest h-index, and Fundacao Oswaldo Cruz is the most productive institution. (4) Brazil, India, the United States, the United Kingdom, and the Netherlands are the most productive countries, and the collaborative network reveals that they have established close cooperation with other countries. France has the highest average number of citations. (5) The keyword co-occurrence network identifies five highly relevant clusters representing topical issues in leprosy research (public health, leprosy vaccine, immune mechanisms, treatment, and genomics research). Overall, these results provide valuable insights for scholars, research institutions, and policymakers to better understand developments in the field of leprosy.

## 1. Introduction

Leprosy is a chronic infectious disease caused by Mycobacterium leprae, and skin lesions and peripheral neuropathy are its main clinical features [1]. However, its clinical presentation is unfamiliar to most patients, and the associated immune response may be similar to other more common diseases, such as systemic lupus erythematosus [2]. It has led to misdiagnosis, delayed treatment, and irreversible neurological damage [3].

Since the introduction of multidrug therapy (MDT) in the last century, the treatment of leprosy and the outlook for patients have improved dramatically [4]. Some studies revealed that the global leprosy burden mainly affects developing countries. Brazil, India, and Indonesia reported the most cases annually, accounting for approximately 50% of the reported cases [5,6]. In contrast, the number of cases reported in developed countries tends to be much lower, such as Australia, where only four cases were reported in 2020 [7]. The disease can be cured, but it remains a significant health problem. For example, a prospective survey of available data from endemic countries shows a global drug resistance rate of 8% in nearly 2000 reported cases, which poses a significant problem for leprosy patients who have been taking medication for a long time [8,9]. The stigma attached to the disability of leprosy patients is also an important public health issue. A study conducted in Ghana reported that people who recovered from leprosy preferred to stay in leprosariums instead of returning to society. It is due to isolation, self-stigma, and neglect, which can render effective treatment for leprosy irrelevant [10]. In conclusion, despite the decline in new diagnoses, complications arising from leprosy continue to plague the health of patients.

The increase in the number and scope of publications on leprosy may pose new challenges to scholars. The trends, research hotspots, and research frontiers in leprosy are not clear. In summary, a comprehensive literature review is essential for scholars to understand current research advances and identify possible research directions. Bibliometrics is a tool for assessing trends and hot spots among published papers and research areas through mathematical and statistical methods. It provides objective data on scientific results in different fields (e.g., mathematics, radiology, biology, or hypertension) [11,12,13,14]. The assessment makes it possible to compare the performance of scientific outputs between different countries, institutions, and authors. For instance, WT Yang used bibliometrics to provide a systematic view of infectious disease forecasting, which is helpful for scholars to assess the characteristics of publications and for policymakers to adopt scientific responses [15].

This study used analytical tools to conduct a bibliometric analysis of leprosy publications stored in the Web of Science database between 2000 and 2021. The analysis focused on the following points: (1) General publication trends and subject categories were described to help the researcher understand the current status of leprosy publications. (2) The journals with the highest number of leprosy publications were collected to select appropriate journals for researchers to publish in. (3) Identify the countries and institutions involved in leprosy research and make researchers aware of international and potential collaborators. (4) Discuss topical issues during the research period to inform researchers’ choice of research directions.

## 2. Methods

### 2.1. Database Sources

The Web of Science(WOS) core Collection database is a selective citation index for scientific and scholarly publications, including journals, conference proceedings, books, and data compilations, and it is developed by Eugene Garfield of Clarivate Analytics (version2022, Boston, MA, USA). Wos is probably the most commonly used database for bibliometric analysis as it provides robust web-based bibliographic and citation data covering over 12,700 highly rated and influential journals worldwide.

### 2.2. Searching Strategy

“Leprosy,” “Hansen Disease,” and “Hansen’s Disease” were selected as keywords for input into the search engine so that publications with these search terms in the abstract, keywords, or titles could be found. Document retrieval and recording were completed on 22 March 2022 to avoid changes in citations caused by frequent database updates. In order to improve the quality of the search, we have adopted an advanced search function with the following search rules: Document types = ‘All types;’ Time span = ‘1 January 2000–31 December 2021;’ Languages = ‘All languages;’ Database = SSCI, SCIE in WoS Core Collection.

We further grouped publications from England, Scotland, Wales, and Northern Ireland as publications issued from the United Kingdom (UK), while publications from mainland China, Hong Kong, Macao, and Taiwan were grouped separately. Global collaboration depends on whether co-authors are from more than one country.

### 2.3. Data Extraction and Statistical Analysis

The WOS database has a built-in statistical tool (Incites) that shows trends in publications by year and the distribution of journals, countries, institutions, etc. Impact Factor (IF) values were collected by the Journal Citation Reports (JCR) in 2020. All the results can be found in WOS. For this study, the figures of journals, countries/regions, authors, citations, h-index, and annual publications were plotted by the original 2021 (Origin-Lab Corporation, Northampton, MA, USA). Tables were summarized by using Microsoft Office Excel (Microsoft Corp, Redmond, WA, USA). We have also analysed the collaboration between authors, institutions, and countries/regions by using Vosviewer 1.6.6 (Leiden University, Leiden, The Netherlands) software. In addition, the map of national collaborations was created by using Scimago Graphica software (Version1.0.17, Scimago, Granada, Spain, (https://www.graphica.app/ (accessed on 25 March 2022)).

## 3. Results

### 3.1. Types and Categories 

**Figure 1:**
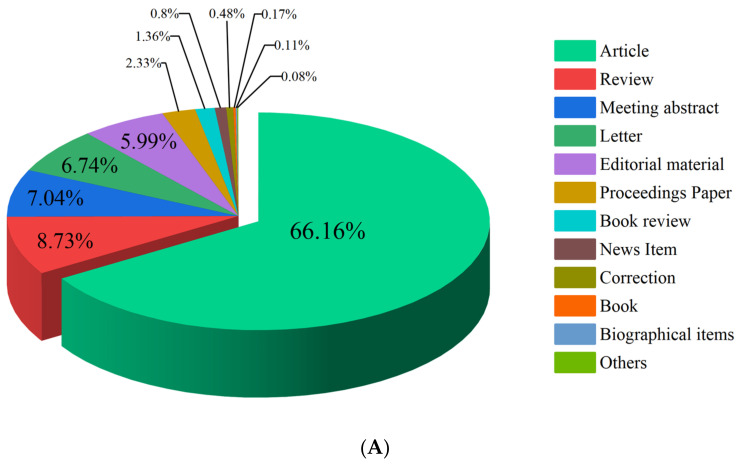

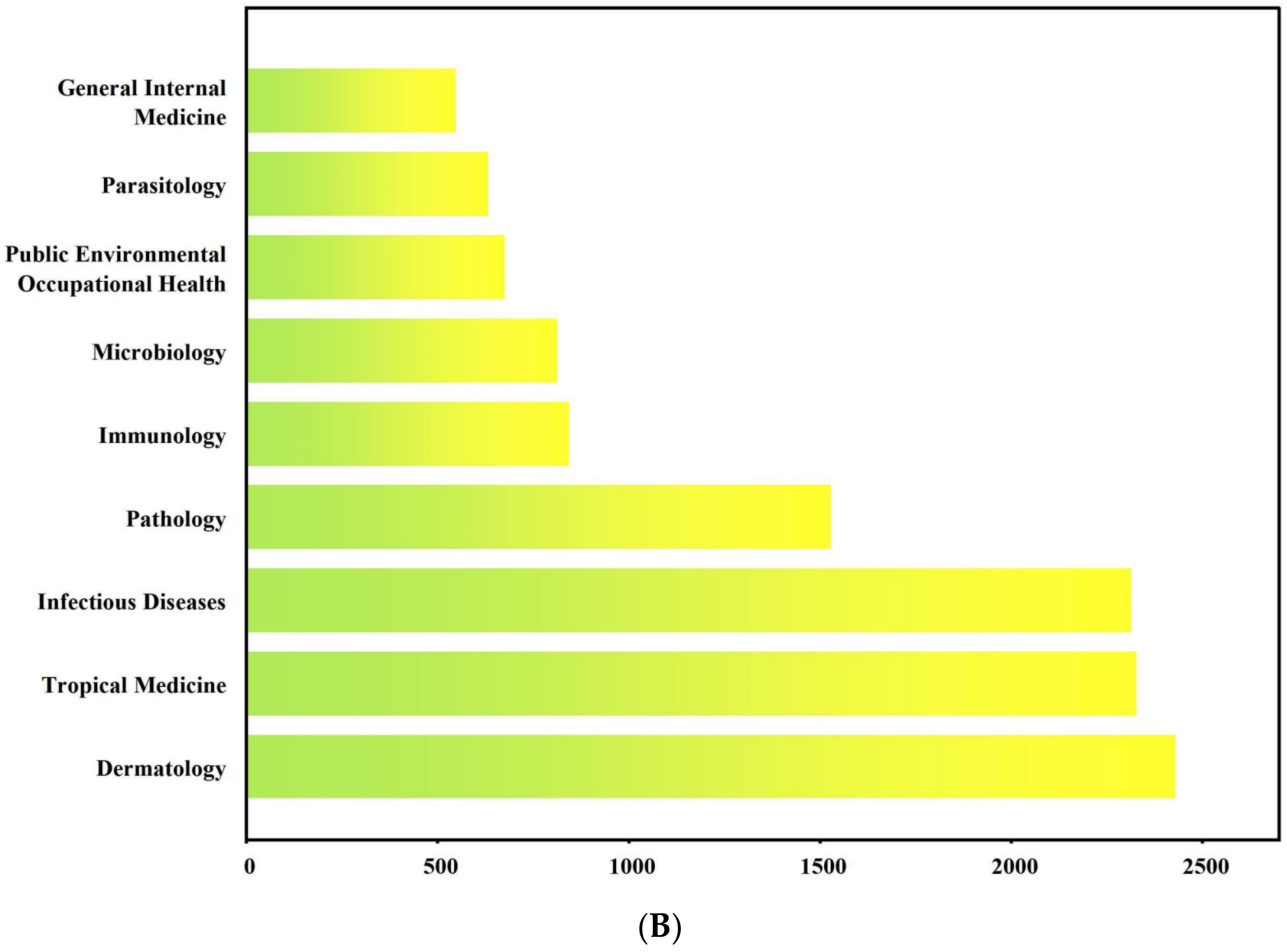
(**A**) Types of publications in the field of leprosy (2000–2021). (**B**) The top 10 subject categories in the field of leprosy (2000–2021).

Figure 1A shows the distribution of the publication types. Research papers were the most common type of publication, accounting for 66.16% of all publications, followed by review (8.73%), meeting abstract (7.04%), letter (6.74%), editorial material (6.01%), proceedings paper (2.33%), book review (1.36%), news item (0.80%), and others. The publications were available in 12 languages, of which 95.13% were English. Other languages included Portuguese (1.73%), Spanish (1.02%), French (1.01%), and German (0.71%).

There is a great diversity of research topics on leprosy, with 131 subject categories identified. Figure 1B shows the ten subject categories with the highest number of publications. The main subject category covered by these publications was “dermatology (2430),” followed by tropical medicine (2327) and infectious diseases (2314).

### 3.2. Publications Statistics

**Figure 2:**
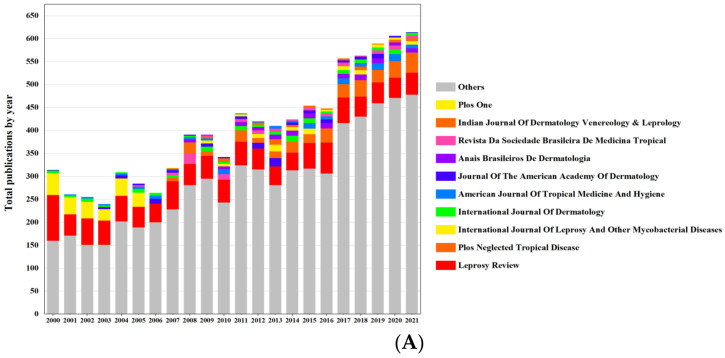

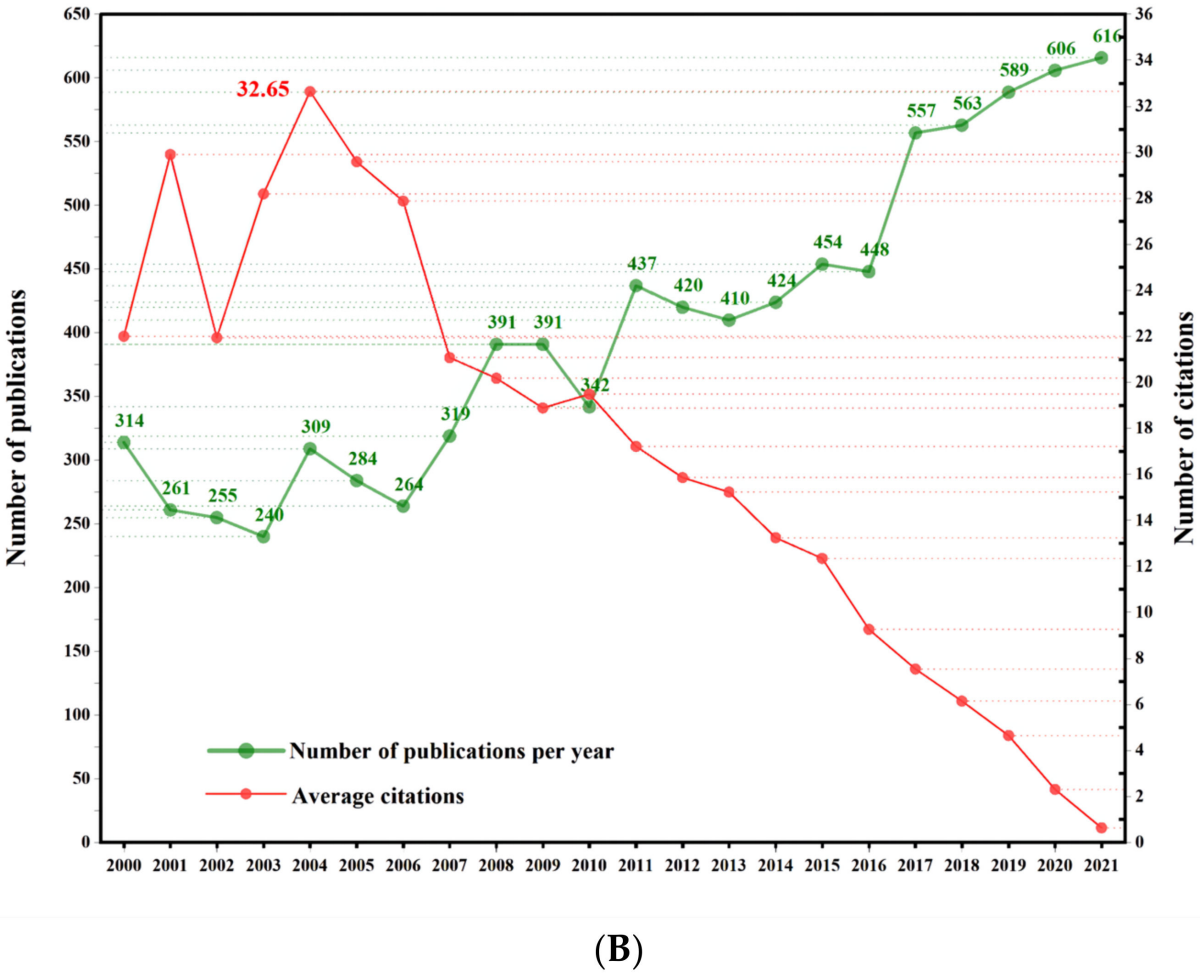
(**A**) Number of publications and citations in the field of leprosy (2000–2021). (**B**) The performance of the 10 most productive journals in all the publications (2000–2021).

We obtained 8892 papers for this study. Figure 2A shows the evolutionary trajectory of the number of papers and citations on leprosy. The publications increased steadily from 2000 (*n* = 314, 3.53%) to 2021 (*n* = 616, 6.93%). Nevertheless, a decreasing trend was found in the number of publications from 2000 to 2003 (314 to 240). The average number of citations for all the publications was 14.56, and the highest average citation frequency (32.65) was observed in 2004.

Research related to leprosy was published in 1913 journals, with 39 journals publishing over 30 papers. As shown in Table 1, “*Leprosy Review*” (IF = 0.537) was the most productive journal, with 1148 related papers. It covered leprosy’s medical, physical, and social aspects and relevant information on leprosy control, followed closely by “*Plos Neglected Tropical Disease*,” which contributed 3.48% to the overall publications. “*Journal of the American Academy of Dermatology*” was the highest impact factor of the ten journals (IF = 11.527), which published 122 papers (1.37%). In addition, we visualized the annual publication of the ten most productive journals in the field of leprosy (Figure 2B).

### 3.3. Author Analysis

The ten most productive authors of leprosy-related publications are listed in Table 2. Leprosy-related research has been contributed to by 24,672 authors. Three authors have published more than 100 papers. Sarno, EN from the Oswaldo Cruz Foundation contributed the most papers (199, 2.24%), followed by Richardus, JH from the University of Wisconsin–Madison with 153 (1.72%) publications. Lockwood, DNJ, from the London School of Hygiene and Tropical Medicine, published more than 100 papers (115). In addition, Hong Liu and Furen Zhang from China published 47 and 39 papers, respectively, ranking 32nd and 45th during the study period.

**Figure 3:**
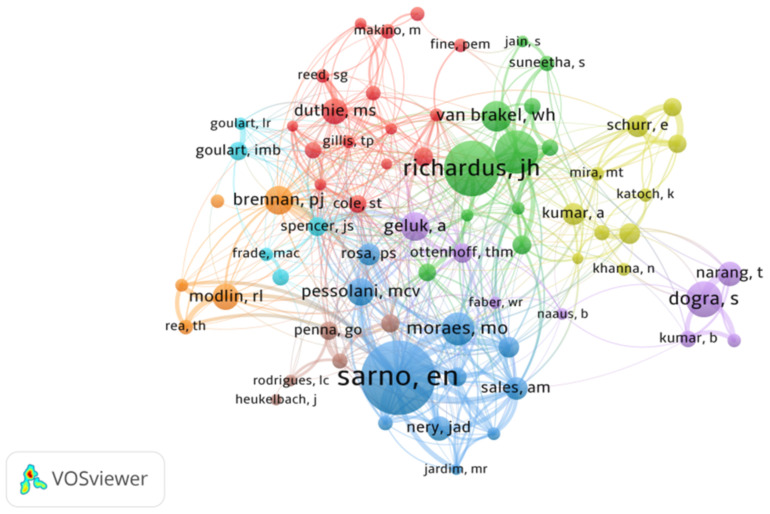
A collaborative network of co-authors in the field of leprosy (2000–2021).

The co-authorship network in the field of leprosy is shown in Figure 3. There were eight co-authorship clusters. The size of the circle is proportional to the number of papers published by the author, the color of the circles corresponds to the year of publication, and the thickness of the lines is proportional to the frequency of collaboration. For example, Sarno EN links to Moraes MO, demonstrating a partnership between them. The thickness of the lines between them also testifies to the degree of cooperation, several large collaborative clusters, and several smaller ones, such as the orange collaboration network between Chinese scholar Hong Liu and US scholar Brennan PJ.

### 3.4. Institution Analysis

**Figure 4:**
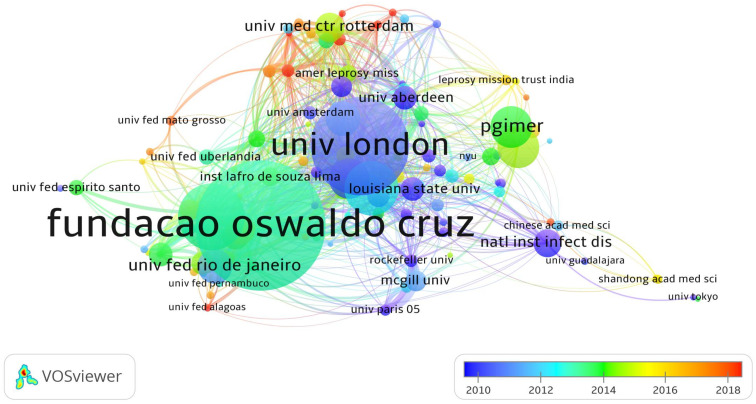
A collaborative network of institutions in the field of leprosy (2000–2021).

Among the organizations identified from the bibliographic data, 117 organizations have published more than 20 papers. As shown in Figure 4, the color of the circles is opposed to the year, the size of the circles is proportional to the number of documents arranged, and the thickness of the lines is proportional to the frequency of cooperation. The figure also shows that these institutions have collaborative relationships with most of the influential scientific institutions in leprosy research. It can also be seen that the University of London was one of the first institutions to start collaborations with other countries during the period (including the London School of Hygiene and Tropical Medicine).

Table 3 presents the ten most productive institutions, their geographic location, and the number of publications. The three most productive organizations were Fundacao Oswaldo Cruz (480), the University of London (425), and the Universidade de Sao Paulo (249). The University of London had the highest h-index (56), followed by Fundacao Oswaldo Cruz (45) and the University of California System (38). The ten institutions were affiliated with Brazil (3), the United States (2), the Netherlands (2), India (1), and the United Kingdom (1).

### 3.5. Country Analysis

**Figure 5:**
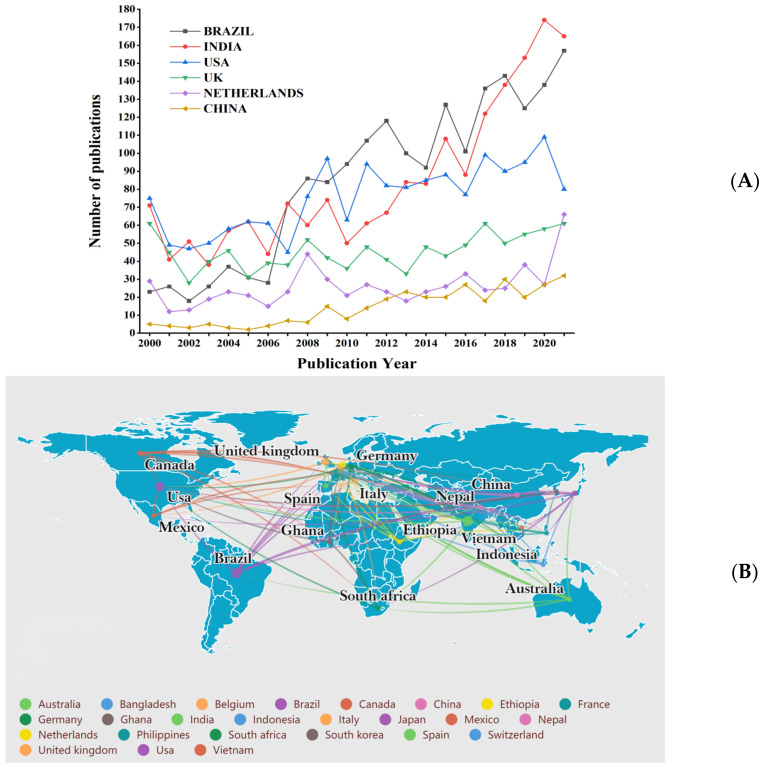

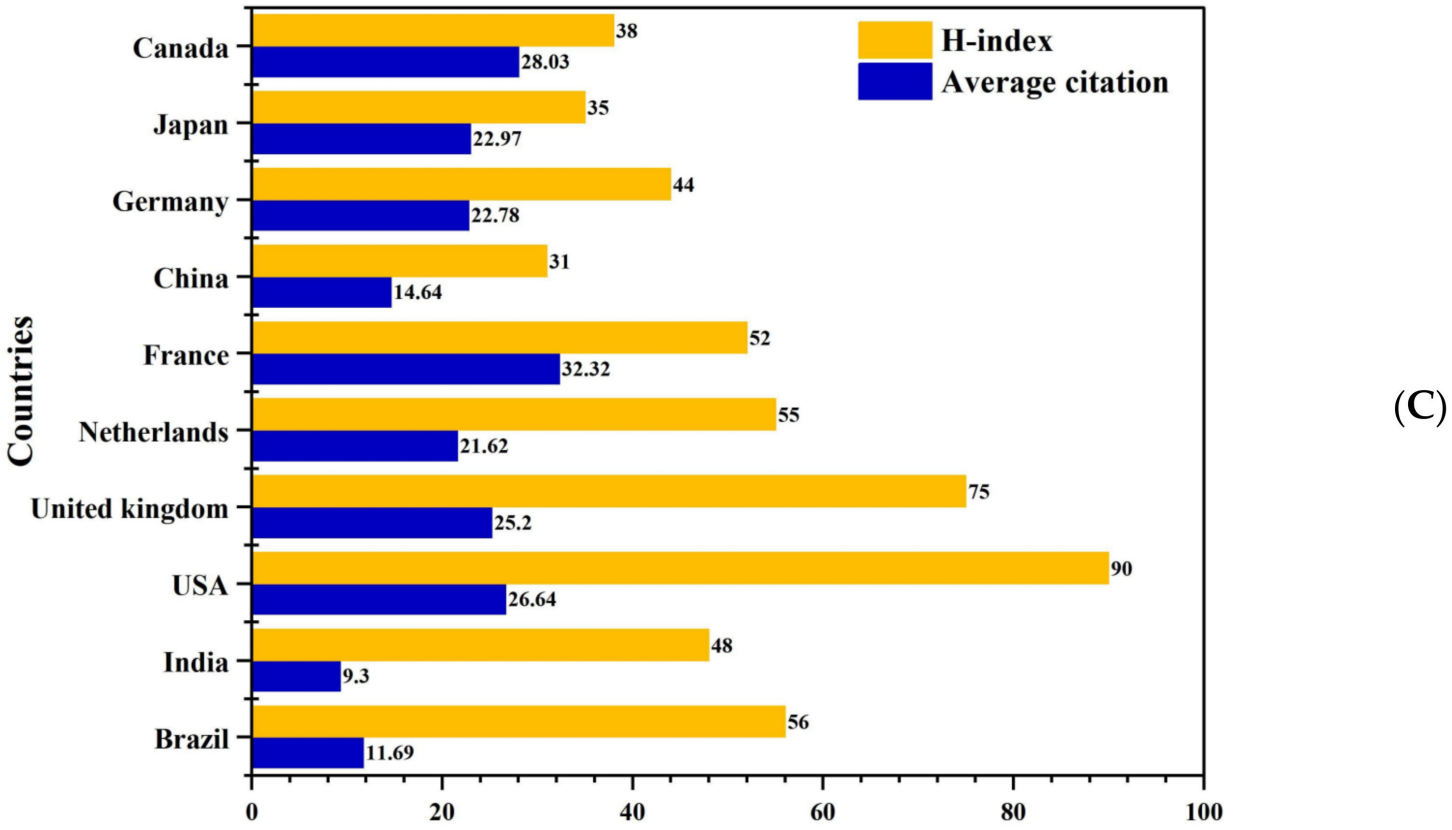
Cooperation between countries/regions in the field of leprosy (2000–2021). (**A**) The number of publications for the top 5 countries related to leprosy. (**B**) Map of inter-country cooperation on leprosy (2000–2021). (**C**) H-index and average citation rate per paper for the ten most productive countries.

As shown in Figure 5A, the output of Brazil, India, the USA, the United Kingdom, and the Netherlands increased during this period. Brazil was the country with the highest paper production (1869, 21.01%), followed by India (1863, 20.95%), the USA (1663, 18.70%), the United Kingdom (893, 10.04%), and the Netherlands (580, 6.52%). Publications output in the UK and the Netherlands has remained relatively stable. The growth in publications production from China was notable, which increased from just two papers in 2000 to 32 papers in 2021.

Figure 5B illustrates the level of cooperation between countries; 151 countries and regions participated in the publication of these papers, with five countries publishing over 400 papers and 19 countries publishing over 100 papers. We used Scimago Graphica software (Version 1.0.17, Scimago, Granada, Spain) to visualize the VOS viewer’s network data to get a more intuitive view of the cooperation between the different countries. Each dot represents a country, and the size of the connected dots represents the strength of collaboration. The strength of cooperation is indicated by the thickness of the connection between the countries. Cooperation networks have been established in Brazil, the United States, India, and the United Kingdom.

Figure 5C depicts the h-index and average citation counts of papers in the top ten countries. The top five countries in the h-index are as follows: the USA (90), the UK (75), Brazil (56), the Netherlands (55), and France (52) round out the top five. Meanwhile, France (32.32), Canada (28.03), and the USA (26.64) were the top three countries with the highest average number of citations. In addition, China performed poorly in the h-index (31) and average citation counts (14.64).

### 3.6. Keyword Analysis 

In this study, equivalent keywords written in different ways were identified, such as “Hansen’s disease” and “Hansen disease” or “erythema nodosum leprosum” and “erythema nodosum leprosum.” We have also analyzed the occurrence of the keywords frequently used among the top five countries and China (Table 4). The most common keywords were “mycobacterium leprae,” “leprosy,” “diagnosis,” “disease,” “infection,” “leprosy,” and “Mycobacterium leprae” were the most frequently used keywords in the top five countries. In addition, the keywords “association (22),” “identification (21),” “diagnosis (21),” and “susceptibility (20)” received more attention in the papers from China.

**Figure 6:**
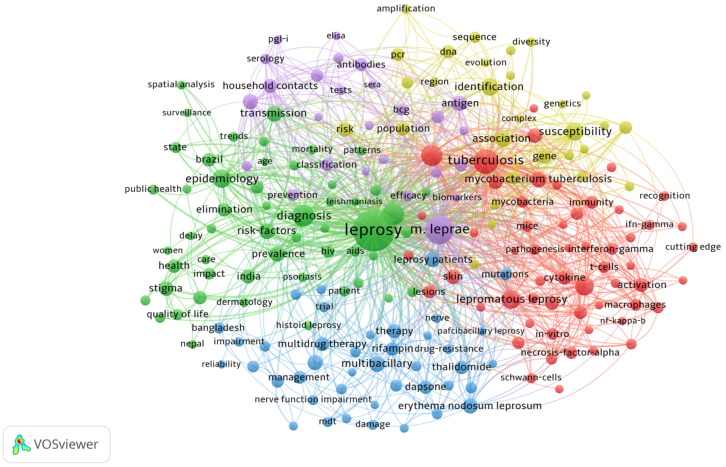
Keyword co-occurrence network about the field of leprosy (2000–2021).

As shown in Figure 6, five themes of leprosy research were found. The green cluster is involved in public health (quality of life, diagnosis, disability, stigma, spatial analysis, etc.). The yellow cluster primarily concerns the leprosy vaccine (household contacts, infection, BCG, etc.). The purple group is mainly about genomics research (amplification, PCR, evolution, sequence, etc.). The red cluster dealt with the immunological mechanisms (macrophage, immune response, cutting edges, etc.). The blue cluster is mainly concerned with the treatment of leprosy (leprosy patients, multi-drug therapy, drug resistance, relapse, etc.).

## 4. Discussion

In recent years, after the essential elimination of leprosy (of which there is a prevalence of <1/100,000), the trends, research hotspots, and research frontiers about leprosy research are not clear. We set the period of 2000–2021 as the study node to explore the publication characteristics of leprosy-related publications and provide new ideas for scholars.

Furthermore, 24,672 authors have published 8892 papers in 1913 journals. The number of publications, cooperation between countries, and their contributions to leprosy have all increased. However, a decreasing trend was found in the number of publications from 2000 to 2003 (314 to 240). It may be due to the advent of multi-drug therapy and anti-inflammatory treatment adopted by the WHO, which led to a significant reduction in new cases of leprosy at the time, thus reducing the research fervor in leprosy [16,17].

With the exception of the “Journal of the American Academy of Dermatology” (IF = 11.53), the ten most productive journals all have low impact factors. For example, the top- ranked journal “Leprosy Review” has published 1148 papers, but its impact factor was only 0.537. The low impact factor of these journals reflects the reality of a lack of innovation in leprosy research [18]. Brazil, India, and the USA have the highest number of leprosy publications, accounting for 56.53% of all the publications, and have developed a strong collaborative network. The results demonstrated that leprosy research is more valued in developing countries with high prevalence rates [19,20]. The United States displays its leadership in leprosy research through a comprehensive assessment of the h-index, citations, publication output, and international collaborations. It was also the leader in various areas, such as stigma, nasopharyngeal cancer, and suicide [21,22,23]. India has published many papers (1863), but its h-index and the average number of citations are low due to the limited resources and funding available to support the publication costs of high-quality journals. France has the highest average number of citations (32.32). Only very few papers led to the high citation of publications from France. For instance, French scientists used genetic analysis to reveal the origin of the leprosy bacterium and how it spreads with human migration, which provides essential clues for understanding the etiology of leprosy [24].

We have analyzed the occurrence of keywords used in the top five countries.” Mycobacterium leprae,” “leprosy,” “diagnosis,” “disease,” and “infection” are highly cited keywords. As a leading country in leprosy research, these keywords are also highly cited in the USA. The keyword “diagnosis” revealed an excellent phenomenon that when patients are diagnosed earlier and treated better, disabilities and stigma will disappear. Future research should go into this. “Association” is more cited in China. It may be because Chinese scholars are more interested in the relationship between genes and leprosy, and they have published some highly rated papers [25,26].

We also identified five highly relevant clusters through a keyword co-occurrence network, representing the topical issues in leprosy research. The hotness of the network clusters is sorted by serial number size: (#1) Public health: In this cluster, the priorities for leprosy studies include the need to create accurate methods to detect individuals who are in subclinical infection and diagnose new cases correctly (especially in the early stages of the disease). If the clinicians diagnose early and particularly treat them well, there will be no disabilities and concerns about the quality of life [27]; and to provide ongoing professional health training to improve patients’ quality of life and develop campaigns to raise awareness of the disease and reduce stigma [28]. There has been a growing body of research on “stigma” in recent years, which may raise eyebrows in the future. Some experts indicated that it is a bad sign that the focus has shifted from clinics and the understanding of the disease to stigmatization [29,30]. The previous studies have shown that leprosy is spatially aggregated, and there is a growing body of studies applying GIS to leprosy, particularly in Brazil, India, and China [31,32,33]. (#2) Immune mechanisms: Innate immune cells in leprosy are one of the focuses of immune mechanisms [34]. Macrophages, Schwann cells, dendritic cells, and keratinocytes are the study’s main cell populations of interest. As for macrophages, it has been proven that bone marrow-derived monocytes enter the tissues in large numbers and differentiate into M1 and M2 phenotypes of macrophages under regulation, which regulates infection by Mycobacterium leprae [35]. In addition, the application of cutting-edge technologies to the study of immune mechanisms in leprosy is also one of the popular research directions (e.g., single-cell RNA sequencing) [36]. (#3) Treatment: Drug resistance is an important consideration in treating leprosy. According to the recommendations of the World Health Organization, multi-drug therapy was adopted as the main treatment for leprosy (Rifampicin, Aminophenazone, and Clofazimine) [37,38,39]. However, there have been increasing reports of drug-resistant strains of Mycobacterium leprae, and the local drug-resistance rate has reached 8%. Some studies revealed that clinical manifestations after MDT may recur due to leprosy reactions, inadequate treatment, or reinfection [40]. The inability to culture Mycobacterium leprae in vitro also limits effective testing for drug resistance in recent studies. (#4) Leprosy vaccine: Chemoprevention of leprosy in susceptible populations (e.g., household contacts) has been relatively successful. However, chemoprophylaxis does not ensure that an exposed person will not develop the disease upon exposure to Mycobacterium leprae [41]. Vaccines are often seen as an essential tool in eradicating infectious diseases, so a specific vaccine capable of inducing a lasting immune response is one of the most important means of preventing infection. BCG vaccination has already proven to be an excellent preventive measure against leprosy [42]. Nevertheless, it has less favorable effects, such as an increase in paucibacillary leprosy patients during the first few months after vaccination and re-vaccination, and the vaccination strategy is not supported in the WHO guidelines. Thus, developing leprosy-specific vaccines that promote long-lasting T-cell responses is more promising [43]. (#5) Genomics research: In this cluster of leprosy research, scholars have focused on molecular tools and genotyping, strain evolution, animal reservoirs, the relationship between strains/genotypes and disease characteristics, etc. For instance, genotyping procedures have been developed for PCR sequencing and the genome-wide analysis of Mycobacterium leprae bacilli. It provides the foundation for molecular epidemiological studies to understand better the evolution of strains associated with ancient human migrations and phylogeographic insights on the spread of disease. There are still many challenges in the study of leprosy genomics. For example, the exact mechanisms of transmission remain unclear; the virulence and transmission rates of leprosy are difficult to measure; the complete genomes of rare genotypes have not been sequenced; and the sampling and genome sequencing of isolates from different regions (e.g., Indonesia, South Asia, and the Middle East) are inadequate [44,45].

Recently, WHO announced its new strategy toward zero leprosy with the Global Leprosy (Hansen’s Disease) Strategy, 2021–2030 [46]. Some specialists indicated that closer cooperation between countries/individuals is the best way to achieve the goal, while more high-quality papers should be published to provide references for the plan toward zero leprosy. Based on the study results, the following points should be noted to improve innovation in leprosy: Firstly, strengthen multidisciplinary intersections in the field of leprosy. For example, combining traditional epidemiology with archaeology, geography, public policy, or intelligence informatics; Secondly, enhance national attention to leprosy research and increasing financial investment by the government, especially in high endemic countries; Thirdly, cultivating professional leprosy research-oriented personnel; Finally, cooperation between countries/individuals should be strengthened, such as between China and India.

There are some limitations to this work. For example, we have limited our publications to the most influential academic databases (WOS). As a result, some papers that may not have been included in the WOS core repository were excluded. However, this work is still representative of the general situation and trends in the leprosy field. We will consider and compare non-English literature from a different platform in the next step studies, such as through Scoups and Pubmed. We confirmed that the paper could benefit clinicians (including dermatologists), medical students, and scientific researchers. It could help them to understand leprosy rapidly and provide helpful information for leprosy-related studies, identifying the trends and potential collaborators. In addition, we hope the clinicians could be more familiar with leprosy after reading the paper and that the number of patients who suffer from leprosy-related sequelae could be reduced in the future.

## 5. Conclusions

This article analysed research on leprosy published between 2000 and 2021. It provides a detailed overview of leprosy through bibliometrics in papers, journals, languages, years, citations, the h-index, author keywords, institutions, and countries. In fact, it is the first step toward an objective analysis of the existing literature in the field of leprosy research. Brazil, India, the United States, the UK, and the Netherlands play a leading role in global leprosy research production. “Leprosy Review” is the most productive journal. Although newly detected cases have decreased, more countries are involved in leprosy research. Early diagnosis continues to be a research priority in the field of leprosy research.

## Figures and Tables

**Table 1:** The 10 most productive journals for the period from 2000–2021.

Journal	Publications	Percentage (%)	IF	AC
Leprosy Review	1148	12.91	0.54	8.12
Plos Neglected Tropical Disease	309	3.48	4.41	20.36
International Journal of Leprosy and Other Mycobacterial Diseases	214	2.41	0.22	9.95
International Journal of Dermatology	155	1.74	2.74	8.61
American Journal of Tropical Medicine and Hygiene	141	1.59	2.35	10.25
Journal of the American Academy of Dermatology	122	1.37	11.53	4.64
Anais Brasileiros De Dermatologia	119	1.34	1.90	7.22
Revista Da Sociedade Brasileira De Medicina Tropical	112	1.26	1.58	7.85
Indian Journal of Dermatology Venereology & Leprology	97	1.09	2.55	7.72
Plos One	94	1.06	3.24	15.85

AC: average citations.

**Table 2:** The number of publications of the ten most productive authors (2000–2021).

Author	Publication	Percentage	H-Index	Citation
Sarno EN	199	2.24%	35	5059
Richardus JH	153	1.72%	33	2905
Lockwood DNJ	115	1.29%	32	3606
Dogra S	96	1.08%	12	547
Moraes MO	90	1.01%	28	2149
Van Brakel WH	84	0.94%	26	1921
Geluk A	79	0.89%	25	1805
Brennan PJ	77	0.87%	31	2908
Pessolani MCV	73	0.82%	23	1393
Modlin RL	70	0.79%	27	3427
Hong Liu * (32nd)	47	0.53%	13	1285
Furen Zhang * (45th)	39	0.44%	13	1231

* Two Chinese authors.

**Table 3:** The top 10 most productive institutions (2000–2021).

Institutions	Country	Publication/%	H-Index/Rank
Fundacao Oswaldo Cruz	Brazil	480 (5.40)	45/(2)
University Of London	UK	425 (4.78)	56/(1)
Universidade De Sao Paulo	Brazil	249 (2.80)	23/(9)
Indian Council of Medical Research Icmr	India	202 (2.27)	26/(7)
University Of California System	USA	196 (2.20)	38/(3)
Erasmus University Rotterdam	Netherlands	187 (2.10)	34/(6)
CoLorado State University	USA	153 (1.72)	37/(4)
Leiden University	Netherlands	134 (1.51)	36/(5)
Post Graduate Institute of Medical Education Research Pgimer Chandigarh129(PGIMER)	India	129 (1.45)	15/(10)
Universidade Federal do Rio de Janeiro	Brazil	127 (1.43)	24/(8)

**Table 4:** The frequency of keywords used in publications from the five most prolific countries and China, 2000–2021.

China		Netherlands		UK		USA		India		Brazil	
N	Keywords	N	Keywords	N	Keywords	N	Keywords	N	Keywords	N	Keywords
93	leprosy	72	leprosy	138	leprosy	183	mycobacterium leprae	117	leprosy	179	mycobacterium leprae
35	mycobacterium leprae	68	mycobacterium leprae	102	mycobacterium leprae	151	leprosy	84	mycobacterium leprae	144	disease
22	association	48	contact	83	tuberculosis	58	tuberculosis	33	lepromatous leprosy	66	diagnosis
21	identification	41	diagnosis	60	infection	57	diagnosis	33	disease	55	infection
21	diagnosis	35	risk factor	43	disease	53	infection	32	diagnosis	51	risk factor
20	susceptibility	33	tuberculosis	32	identification	52	disease	22	tuberculosis	47	lepromatous leprosy
19	disease	29	infection	31	mycobacterium tuberculosis	40	identification	21	expression	48	leprosy
18	tuberculosis	28	disease	28	diagnosis	35	contact	20	erythema nodosum leprosum	43	susceptibility
16	gene	27	Bangladesh	26	risk factor	32	lepromatous leprosy	19	multidrug therapy	42	association
14	variants	25	chemoprophylaxi	23	lepromatous leprosy	31	antigen	18	childhood leprosy	41	expression

## Data Availability

There are no relevant data for this manuscript other than those presented in the paper.
